# Intravesical Liposome and Antisense Treatment for Detrusor Overactivity and Interstitial Cystitis/Painful Bladder Syndrome

**DOI:** 10.1155/2014/601653

**Published:** 2014-01-15

**Authors:** Pradeep Tyagi, Mahendra P. Kashyap, Naoki Kawamorita, Tsuyoshi Yoshizawa, Michael Chancellor, Naoki Yoshimura

**Affiliations:** ^1^Department of Urology, University of Pittsburgh, PA 15213, USA; ^2^Department of Urology, William Beaumont School of Medicine, Royal oak, MI 48073, USA

## Abstract

*Purpose*. The following review focuses on the recent advancements in intravesical drug delivery, which brings added benefit to the therapy of detrusor overactivity and interstitial cystitis/painful bladder syndrome (IC/PBS). *Results*. Intravesical route is a preferred route of administration for restricting the action of extremely potent drugs like DMSO for patients of interstitial cystitis/painful bladder syndrome (IC/PBS) and botulinum toxin for detrusor overactivity. Patients who are either refractory to oral treatment or need to mitigate the adverse effects encountered with conventional routes of administration also chose this route. Its usefulness in some cases can be limited by vehicle (carrier) toxicity or short duration of action. Efforts have been underway to overcome these limitations by developing liposome platform for intravesical delivery of biotechnological products including antisense oligonucleotides. *Conclusions*. Adoption of forward-thinking approaches can achieve advancements in drug delivery systems targeted to future improvement in pharmacotherapy of bladder diseases. Latest developments in the field of nanotechnology can bring this mode of therapy from second line of treatment for refractory cases to the forefront of disease management.

## 1. Introduction

Intravesical therapies have demonstrated varying degrees of efficacy and safety in treatment of interstitial cystitis/painful bladder syndrome (IC/PBS) [1] and overactive bladder OAB [2]. Pharmacotherapy by this route provides high local drug concentrations in the bladder with low risk of systemic side effects [3]. Conventional therapies for OAB and detrusor overactivity (DO), either neurogenic or idiopathic, have limited efficacy and acceptability. Anticholinergic medications, which are currently the mainstay of the treatment of OAB, are not always effective and often have undesirable side effects such as dry mouth and constipation [4]. Therefore, search for alternative therapies directed against local targets with fewer side effects is encouraged.

The IC/PBS syndrome is characterized by pelvic pain and urinary storage symptoms (e.g., urinary urgency and frequency). The O'Leary-Sant symptom and problem score (interstitial cystitis symptom index (ICSI) and problem index (ICPI)) is recognized as one of the most reliable and valid instruments to identify the extent of bothersome symptoms and the most prominent voiding and painful symptoms in IC/PBS patients [5, 6]. Pentosan polysulfate, PPS, is a synthetic sulphated polysaccharide and is the only approved oral drug for IC/PBS, but it requires daily administration for 6 months before any benefit accrues for the patients [7, 8]. Less than one-tenth of oral dose is excreted into the urine of IC/PBS patients, which is considered to replenish the damaged glycosaminoglycan GAG layer and reduce the influx of potassium back into bladder from urine.

Orally administered agents are often unable to create effective luminal drug concentration due to low urinary excretion of drugs, which justifies bladder instillation. Replacement of GAG layer by intravesical administration of hyaluronic acid has been successfully tried in IC/PBS patients [9]. Intravesical therapy also holds the potential to facilitate the separation of therapeutic actions from side effects by involving a diverse array of novel chemical, pharmacological, and formulation strategies. However, drug delivery by intravesical route is constrained by the impermeability of the urothelium, short duration of action, and the need for frequent administration.

The urinary bladder lining is the most impermeable barrier in the human body [10, 11]. Therefore, therapies delivered directly to the bladder lumen have limited opportunity for systemic distribution, which typically leads to fewer side effects. In addition, the mechanisms for locally delivered therapies can be independent of existing oral therapies, and in some cases, an additive effect can provide compelling comarketing opportunities [12]. We will review the current understanding of urothelium structure and role of intravesical drug delivery in unmasking the pharmacological function of different receptors expressed on its luminal surface.

## 2. Urothelium

Recent investigations have revealed that the urothelium is not just a physical barrier between blood and urine but can express a host of receptors having a functional significance in micturition reflex. The recent identification of a cannabinoid, nicotinic, neurokinin receptors and potassium ion channels in urothelium [13–16] have revealed the role of urothelium as an excitable cell layer in bladder that responds to stretch and convey messages to underlying afferents in bladder. There is also mounting evidence to demonstrate expression of adrenergic, bradykinin, and transient receptor potential (TRP) receptors in urothelium and in proximity of afferent nerves [17, 18]. Urothelium is the primary nonneuronal source for the release of molecules such as adenosine triphosphate (ATP), acetylcholine, and nitric oxide, which are known to affect micturition [19, 20]. Intravesical therapy can be used to unravel the pharmacology of these receptors and paracrine messengers released from urothelium [21, 22].

## 3. Muscarinic Receptors

It is widely accepted that oral antimuscarinics act on muscarinic receptors in the detrusor for managing the symptoms of DO and OAB. Conventional wisdom largely ignores the role of muscarinic receptors expressed on urothelium [23]. Muscarinic receptors expressed on urothelium are believed to be involved in afferent signaling for micturition [23]. The afferent signals are believed to be generated from the basal nonneuronal acetylcholine released during the storage phase from urothelium to enhance the myogenic contractile activity of the detrusor [19, 20].

Theoretically speaking, not only can receptors expressed on the urothelium be influenced by antimuscarinics via the bloodstream, but also few selected antimuscarinics and their active metabolites can affect the muscarinic receptors from the luminal side following their excretion into urine [12]. Alternative mode of action for two antimuscarinic drugs, trospium and solifenacin, was demonstrated by our group [21, 22]. DO in the rat was mimicked by intravesical carbachol [21, 22]. Urine collected from human volunteers who took trospium and solifenacin was then instilled into rat bladder to determine the effect of the drug fraction excreted into urine. Therefore, intravesical therapy can assist in elucidating the yet unexplored mechanisms for improvement of OAB symptoms by antimuscarinics.

## 4. Liposomes

Liposomes were earliest prototype of nanoparticles (particles with one of the dimensions in nanometers) that are described as lipid vesicles composed of concentric phospholipid bilayers enclosing an aqueous interior [24, 25]. The lipid vesicles comprise either one or several aqueous compartments delineated by either one (unilamellar) or several (multilamellar) phospholipid bilayers [26]. Liposomes have been widely studied as drug carriers for a variety of chemotherapeutic agents (approximately 40,000 scientific articles have been published on the liposomes use so far) [25, 27]. Liposomes improved the delivery of chemotherapeutic agents by altering pharmacokinetics and reducing toxicity [26, 28, 29]. The success of liposomes in the clinic has been attributed to the nontoxic nature of the lipids used in their formulation.

## 5. Empty Liposomes

Empty liposomes itself can act as a topical healing agent and same has been demonstrated in treatment of dry eye [30, 31]. Either empty or with entrapped drugs, liposomes have also been used in ophthalmology to ameliorate keratitis, corneal transplant rejection, uveitis, endophthalmitis, and proliferative vitreoretinopathy [32]. These reports encouraged investigation of empty liposomes as a therapeutic agent for bladder injury. Interaction of liposomes with cultured urothelium cells suggested that liposomes can be adsorbed and be endocytosed [33]. Previous studies showed that binding of large multilamellar liposomes to the bladder cells was stronger than of sonicated small size liposomes [34, 35].

### 5.1. Preclinical Studies

**Figure 1:**
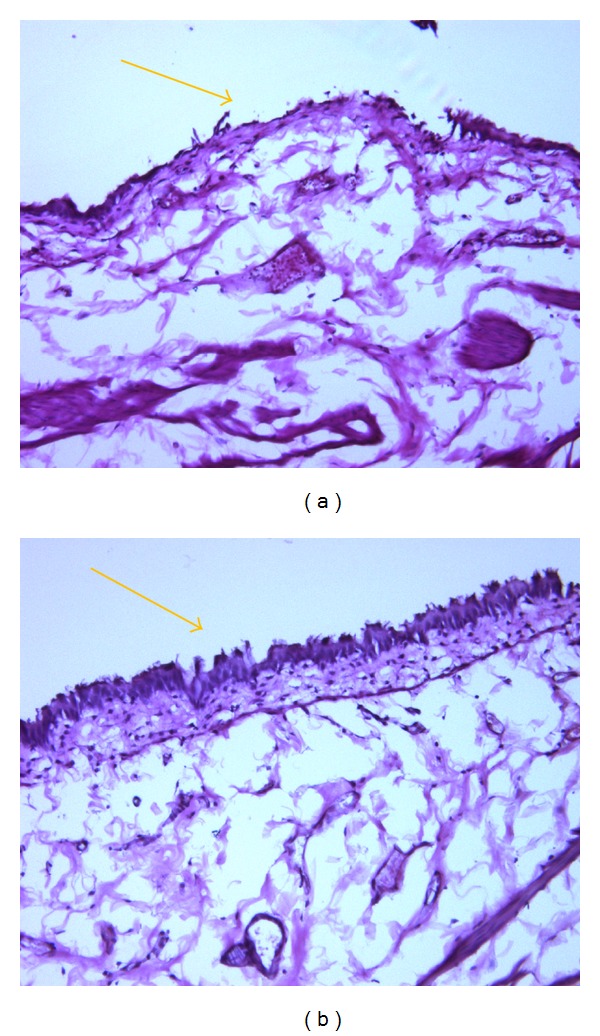
Protective effect of liposomes evaluated by histology in rat bladder injured by protamine sulfate. Focal loss of apical layer and ulcerated areas were noted in saline treated rats (a). Liposome post-treatment protected rats from ulceration induced by PS as evident from photograph in (b) taken on bladder harvest at 8 h after liposome instillation.

A rat model of bladder injury induced by protamine sulfate [36] was used to asses efficacy of empty liposomes. Instillation of liposomes protected bladder irritation induced by protamine sulfate instillation into rat bladder [37] (Figure 1). In earlier study, empty liposomes were used as controls against capsaicin delivery study, which reported tolerance of empty liposomes in uninjured bladder [38]. Bladder tolerance was investigated by cystometry and histology [38].

Physiological effect of liposomes on bladder irritation model induced by protamine sulfate was studied in separate studies [39, 40]. Cystometric studies involved bladder injury induced by infusion of protamine sulfate for an hour followed by irritation caused by infusion of high concentration of potassium chloride solution [39, 40]. Post-treatment of liposomes demonstrated the protective effect in this model [39, 40], which involved coadministration of liposomes with potassium chloride to mimic the clinical disease condition. The comparative efficacy of liposomes was evaluated against FDA approved therapies of dimethyl sulphoxide (DMSO) and intravesical instillation of PPS [1].

Clinically, DMSO (RIMSO-50) is the only FDA approved intravesical treatment for PBS/IC, [41] but off-label instillation of PPS has also been pursued [42]. The efficacy of various treatments was evaluated in chemically induced bladder hyperactivity in rats by sequential infusion of protamine sulfate and potassium chloride. Bladder reflex activity of female Sprague-Dawley rats before and after treatment was evaluated by continuous cystometry under urethane anaesthesia (1.0 g/kg). Intravesical liposomes were effective in doubling the intercontractile interval (ICI) compared with PPS, while acute instillation of DMSO failed to produce any protective effect in this animal model [43].

Recently, Lee et al. [44] further improved this model of DO induced by intravesical infusion by combining it with systemic metabolic alteration through fructose feeding. Metabolic syndrome created by feeding of fructose to rats can lead to DO and urination frequency [44]. Cystometric bladder capacity of fructose fed rats can be further decreased by instillation of acidic ATP solution, which provokes reflex micturition via afferent noise. Evidence suggests that increased expression or release of neurotransmitters in the mucosal layer of the bladder can generate afferent noise via C-fiber pathway and result in DO [45]. Compared to infusion with normal saline, ATP solution decreased bladder capacity and increased phasic contractions. Addition of liposomes to the ATP solution partially reversed the ATP solution-induced response [46]. Capsaicin induced desensitization also blunted the provocation of ATP in this model to demonstrate the role of afferent noise via C-fibers.

### 5.2. Clinical Studies

Encouraged by the exciting preclinical efficacy of empty liposomes as a therapeutic agent for intravesical therapy of IC/PBS, Chuang et al. recently published the clinical safety and efficacy of liposomes in IC/PBS patients [47]. In an open label prospective study on 24 IC/PBS patients, the effect of intravesical liposomes was compared against oral PPS. Patients were equally divided into the two treatment arms, administered either intravesical liposomes (80 mg/40 cc distilled water) once weekly or oral PPS (100 mg) 3 times daily for 4 weeks each. Ten possible responses to treatment were monitored at 3 time points, including baseline, and at weeks 4 and 8.

Comparable efficacy of liposomes to oral PPS was demonstrated by statistically significant decreases in urinary frequency and nocturia in both treatment arms. Liposome treated patients showed statistically significant decreases in pain, urgency, and the O'Leary-Sant symptom score, with the effect being most profound on urgency. None of the treated patients in the study reported urinary incontinence, retention, or infection due to liposome instillation and there were no unanticipated adverse events and no significant worsening of symptoms during followup. Intravesical instillation of liposomes in IC/PBS patients was found to be safe with potential improvement after 1 course of therapy for up to 8 weeks. The study design was suboptimal, with lack of blinding and two treatment arms assigned to different routes of drug administration and regimen. Still once weekly liposome therapy was able to demonstrate efficacy comparable to thrice daily oral administration of PPS.

Subsequently, Lee et al. assessed the safety and efficacy of twice weekly administration of liposomes on IC/PBS symptoms [48]. Five patients were given twice a week treatment of liposomes for 4 weeks. The primary outcome was the change in the O'Leary-Sant symptom/problem score and O'Leary-Sant total score from baseline to week 4 and week 8. The O'Leary-Sant symptom/problem score, O'Leary-Sant total score, and pain score at the 4-week followup showed significantly greater improvement from baseline with biweekly instillation than once a week instillation. Tolerability of liposomes remains unchanged from once a week regimen to twice a week regimen. The followup at 8 weeks was also similar for both treatment regimens. The incidence of urinary incontinence, retention, or unanticipated adverse changes was not noted with any regimen. Intravesical liposomes appear to be a promising new treatment for IC/PBS and future large-scale placebo controlled studies are needed to verify these results from a pilot study.

**Figure 2:**
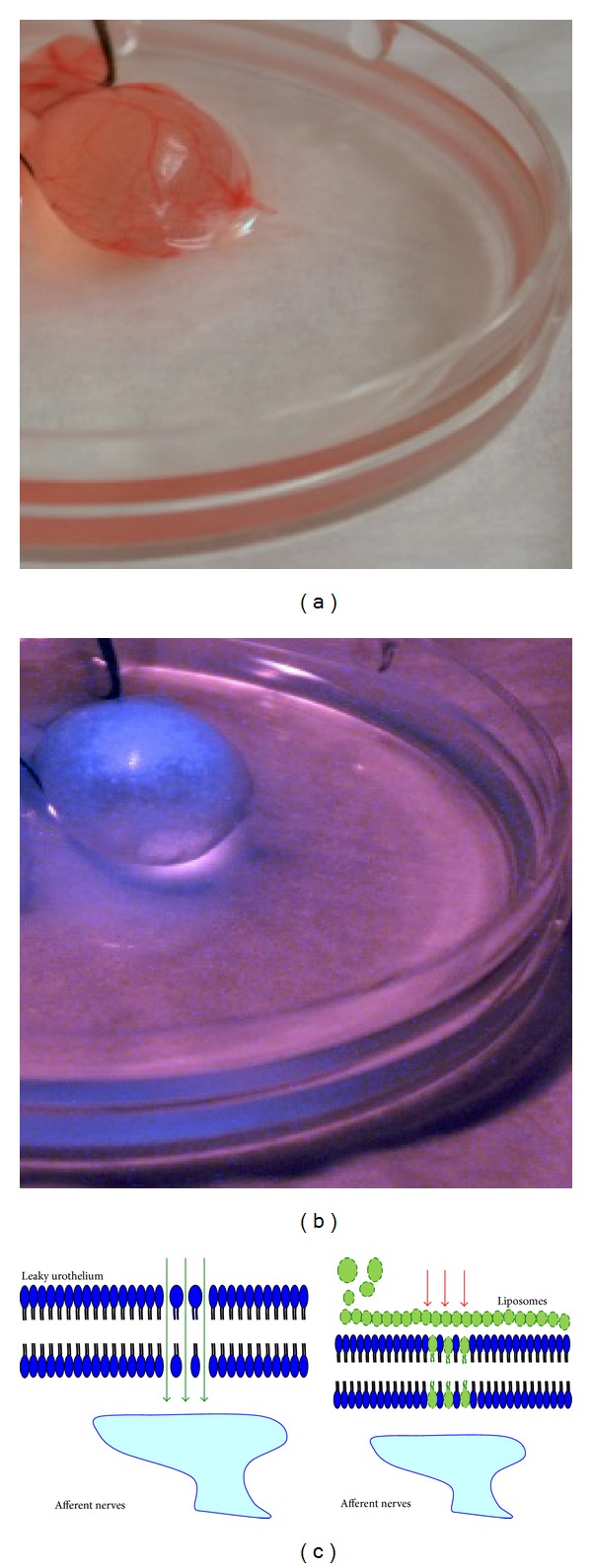
*Ex vivo* images of rat bladder taken in visible and near-infrared light indicate the coating formed by instilled liposomes on bladder surface. Liposomes carry a trace amount of near-infrared (NIR) lipophilic carbocyanine dye 1,1′-dioctadecyl-3,3,3′,3′-tetramethylindotricarbocyanine iodide (DiR) that fluoresces on exposure to NIR light. Rat bladder filled with urine was tied at the base with thread prior to harvest. Liposomes coating the bladder surface are invisible in visible light photograph (a) but is indicated by blue colored coating on the bladder luminal surface in NIR light (b). NIR imaging *in vivo* can allow noninvasive repeat objective measurement for bladder residence time of instilled treatments. (c) Schematic illustration of liposome coating the bladder surface. Given the chemical affinity of phospholipids of instilled liposomes with lipids in cells lining the bladder surface, the liposomes form a protective film coating on the injured bladder lumen surface and assist in the repair of leaky and inflamed uroepithelium.

The exact mechanism of action for liposomes in IC/PBS remains to be established, but protective coating effect based on preclinical studies cannot be ruled out as illustrated in Figure 2.

## 6. Liposomes as a Delivery Platform

Not only is the lining of the urinary bladder the most impermeable tissue in the human body, but it is also the most compliant. As a bladder lining expands, additional membrane material is added to its cells to help retain impermeability [49]. Therefore, vesicular trafficking may provide a favorable environment for drug delivery and therefore it is worth investigating whether vesicle nature of liposomes can aid in improving the delivery of cargo across the bladder permeability barrier.

In the field of neurourology, instillation of neurotoxins into bladder is an accepted approach to achieve chemical neuromodulation of afferent neurotransmission underlying neurogenic bladder and IC/PBS [50]. Existing approaches of chemical neuromodulation by intravesical neurotoxins are suboptimal due to vehicle toxicity for capsaicin [51] or degradation of botulinum toxin (BoNT) in urine. Possible reasons underlying the lack of efficacy from BoNT instillation in bladder can be protein degradation by proteases and proteinases in urine, dilution in urine, or poor uptake of the BoNT solution into the urothelium.

**Figure 3:**
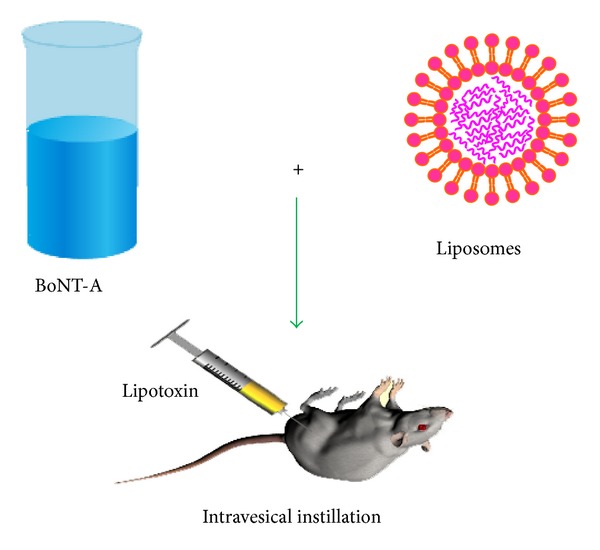
Scheme for formulation of BoNT into liposomes for bladder instillation. Freeze dried BoNT is resuspended in distilled water and added to vials containing a desiccated liposomal preparation made of phospholipids. This reconstituted suspension is allowed to hydrate at room temperature for 30–60 min before instillation.

Liposomes have been previously studied as a carrier of toxins to enhance efficacy at lower doses [52]. In the context of toxins instilled in the bladder, fat-soluble neurotoxin such as capsaicin can be integrated into the phospholipid bilayer [38] and water-soluble neurotoxin such as BoNT can be protected inside the aqueous compartment(s) of liposomes delimited by the phospholipid bilayer(s) [53] (Figure 3). Cystoscope guided injections are the current standard practice in the clinic for administering BoNT to the bladder. But in recent years, studies have also assessed the potential for intravesical instillation of BoNT alone in animal models of bladder irritation [54].

Development of instillation as a mode for administering BoNT in patients will drastically bring down the cost of treatment for patients with refractory overactive bladder. Other groups have recently reported the use of DMSO for instillation of BoNT instead of injection [55]. DMSO does not afford the natural state of the BoNT as a protein and need to be formulated immediately before instillation. Dissolving BoNT in DMSO may not be advisable owing to concerns of BoNT uptake into the systemic circulation of patient. Moreover, biochemical studies have demonstrated that metalloproteolytic activity of the BoNT is strongly enhanced by the presence of lipid membranes [56].

Recent studies reaffirmed the potential of liposomes as a promising vehicle for delivery of neurotoxins to the bladder [38, 53]. The transport of BoNT into urothelium from liposomes was confirmed by detection of its unique effect on neurotransmitters and proteolysis of synaptosomal-associated protein SNAP-25 through immunohistochemistry [53]. The protection of BoNT entrapped inside liposomes from degradation in urine without compromising efficacy was demonstrated by attenuation of acetic acid induced bladder irritation in rats [53]. Similar results were obtained in preclinical studies with liposomes encapsulating capsaicin [38]. Liposomes have proven themselves as biocompatible delivery agents in the bladder.

## 7. Intravesical Antisense Therapeutics

The term “antisense” therapeutics emerged from seminal studies done 4 decades using a short synthetic oligonucleotide for sequence-specific gene silencing [57]. Gene silencing involves introduction of short strands of DNA (termed as antisense) with sequences complementary to the mRNA encoding a particular gene inside the cell with the intent to block gene expression through either translational inhibition or enzymatic cleavage of the mRNA target [3].

**Figure 4:**
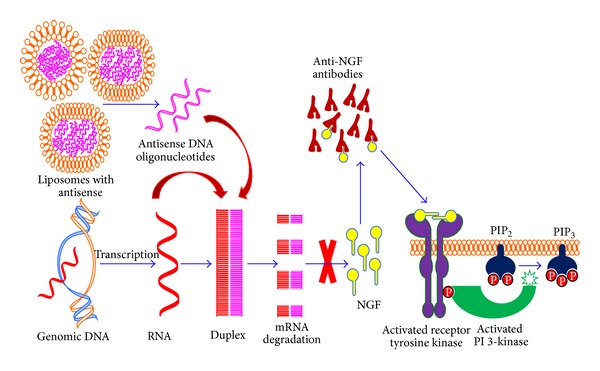
Illustration of mechanisms for blocking NGF expression by either NGF antibodies or downregulation of NGF mRNA expression. NGF antibody sequesters the freely available NGF and prevents it from binding with the cognate TrkA receptors. NGF antisense selectively binds with NGF mRNA inside the cells to block the translation of corresponding mRNA. siRNA takes advantage of endogenous cellular pathways to degrade NGF mRNA and control the posttranscriptional NGF gene expression.

Oligonucleotide ODN binds specifically and strongly to the mRNA target through Watson-Crick base pairing. ODN can be basically categorized into those that direct cleavage of the target mRNA as caused by small interfering RNAs (siRNAs) and those that alter mRNA translation without causing mRNA cleavage. Recent discovery of small interfering RNAs (siRNAs) and the elucidation of the RNA interference (RNAi) pathway has also brought a sea change in the control of posttranscriptional gene expression. siRNA takes advantage of endogenous cellular pathways to potently silence the expression of specific genes (Figure 4).

Antisense mechanism is a promising approach for developing therapeutics based on rational gene-based drug design. Antisense therapeutics have been under clinical investigation for more than 30 years [58]. However, drug development of this approach has been hampered by inefficient intracellular delivery and cellular uptake of the ODN. The translation of basic antisense research into therapeutics is also impeded by intracellular stability of ODN and potential for “off-target” gene silencing, immunostimulation, and other side effects.

A vast array of chemical modifications to ODN has been developed to overcome the therapeutically limiting features by altering internucleotide phosphate linkages, backbone sugars, or nucleobases. One such modified ODN is peptide nucleic acid, which replaces phosphodiester bond between nucleobases with a peptide bond. Replacement of phosphodiester bond by a phosphorothioate linkage is another method to improve the stability of ODN against nucleases.

Great progress has been made in translating antisense research into clinical therapies based on local injection into eye [58]. The field recently progressed further with a systemic injection therapy for treating familial hypercholesterolemia [59]. Antisense therapeutics have been used for exon skipping to optimize the functionality of a truncated dystrophin protein in dog model of Duchenne muscular dystrophy [60].

Nevertheless, applied research for bladder diseases has lagged behind, considering that the anatomical architecture of bladder provides ease of local administration with restricted systemic side effects due to lower serum uptake of antisense ODN. Bladder instillation of antisense ODN or their chemically modified mimics can therefore be an efficient means to control the expression of therapeutically relevant genes. Antisense agents can also be used to elucidate the role of newly discovered genes in bladder function.

## 8. NGF Expression in Bladder

Several studies have reported that patients with OAB or IC/PBS excrete more nerve growth factor (NGF) in their urine relative to asymptomatic controls [61]. Higher expression of NGF in bladder of patients [62], with corresponding lower serum levels of NGF, makes the bladder tissue the likely source for the elevated NGF in urine [63]. Previous studies have indicated that increased levels of NGF in the bladder and bladder afferent pathways are directly involved in the emergence of hyperexcitability of C-fiber bladder sensory pathways leading to the pathology of DO and OAB (Figure 4) [64].

In addition, intrathecal application of NGF antibodies reduced NGF levels in bladder afferent pathways and normalized bladder/urethral function in spinal cord injured (SCI) rats [65]. Because it is likely that the major pathology of OAB is driven by NGF, targeting the intracellular synthesis of NGF molecule in bladder is a promising therapeutic alternative.

## 9. NGF Expression a Drug Target

Overexpression of NGF can be blocked either directly by antibodies [66] or by blocking the synthesis of NGF protein from mRNA [67]. Systemic administration of monoclonal human NGF antibodies (tanezumab) has been explored for therapeutic outcomes in IC/PBS patients but not without encountering safety concerns such as paresthesia, hypoesthesia, and arthralgia [66]. Generalized blockade of NGF activity at sites other than bladder by anti-NGF antibodies may not be the preferred outcome, because NGF is an essential housekeeping growth factor necessary for the survival and growth of neurons [68]. The physiological necessity of NGF action at those sites may explain the incidence of paresthesia, hypoesthesia, and arthralgia in patients treated with systemic anti-NGF antibodies [66].

### 9.1. Peptide Nucleic Acid (PNA)

Therefore, to reduce the toxicity of systemic blockade of NGF, we sought to develop a novel intravesical therapy of OAB by targeting the intracellular synthesis of NGF in the urothelium. Antisense ODN needed to be able to cross cell membrane to act as a drug and negatively charged ODN will not pass through a lipid layer such as cell membranes. Bladder uptake of ODN is limited by the anionic charge and size of the ODN as well as anionic glycosaminoglycan layer of the inner bladder surface. Therefore, primary impediment to be overcome in the development of intravesical antisense therapeutics is inefficient bladder uptake of the ODN across urothelium. Previous studies showed that heparan sulfate proteoglycans expressed on cell surface act as receptors for extracellular TAT uptake [69]. Therefore, it was reasoned that GAG layer on bladder surface can facilitate bladder uptake of peptide nucleic acid PNA, if it could be conjugated with synthetic TAT peptide.

Water insoluble peptide nucleic acid targeting NGF was conjugated with cell penetrating cationic peptide TAT for intracellular delivery of antisense moiety to demonstrate efficacy in animal models. Studies showed that PNA conjugated with TAT suppressed cyclophosphamide cystitis following local instillation of antisense against NGF [67]. There was negligible uptake of peptide nucleic acid in absence of TAT conjugation. Prior to determination of *in vivo* efficacy of conjugate, suitable target sequence on NGF mRNA was determined by predicted folding structure and cell transfection experiments. Successful intravesical delivery of peptide nucleic acid in bladder at the same time of cyclophosphamide injection protected against the cystitis by blocking the rise in bladder contraction frequency and inflammation.

### 9.2. Phosphorothioate-Linked Analogues

Considering the difficulty and possible nonspecific toxicity of peptide nucleic acid and TAT, recent developments focused on simplifying the approach using water-soluble phosphorothioated ODN, which have an increased resistance to exo- and endonucleases for improved stability [70]. As alluded to in the above text, success of antisense therapeutics is largely dependent on the development of a delivery vehicle that can efficiently deliver antisense ODN in bladder. Preliminary studies showed that liposomes can be far better biocompatible effective carriers for local gene silencing of NGF gene in bladder cells.

Cationic liposomes and mimics have emerged as the most popular nonviral method to deliver nucleic acids in therapeutic applications. Easy and reversible complex formation of cationic liposomes with ODN at room temperature allows their use as carriers. Electrostatic attraction between the cationic lipid, DOTAP, and the polyanionic antisense ODN is responsible for the complex formation. The efficacy of liposome delivered siRNA by intravesical route has been previously demonstrated in preclinical models of bladder cancer [71]. Residence of ODN in the rat bladder after intravesical instillation was demonstrated to be longer than 24 h using fluorescent tagged ODN complexed with liposomes [72]. Fluorescent probe was localized in bladder urothelium cells 24 h following instillation. The 24 h residence time was also demonstrated for siRNA in mouse bladder [73]. Bladder uptake of fluorescent ODN without liposomes in normal rat bladder is poor. Previous studies have shown that very high concentration of phosphorothioated ODN can deliver ODN without liposomes to bladder cells of mice having bladder cancer [74]. The urothelium barrier may be slightly compromised in cancerous condition and the strategy of loading bladder with high dose of ODN may not work in noncancerous diseased condition of bladder with intact barrier.

**Figure 5:**
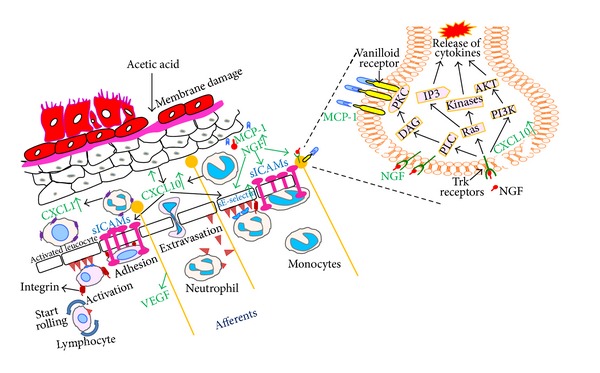
Signalling cascades induced by NGF overexpression following exposure to acetic acid. Injury from exposure to acetic sets off the signalling cascades that upregulate the expression of NGF, MCP-1, prostaglandins, CXCL-1, and CXCL-10. Overexpression of NGF drives the expression of VEGF from neurons and leptin from adipocytes covering neurons. NGF and MCP-1 also induce hyperexcitability in neurons by activating TRPV1 receptor. MCP-1, CXCL-1, and CXCL-10 cause chemoattraction of monocytes, neutrophils, and lymphocytes, which requires extravasation across endothelium from blood through the cooperation of adhesion molecules E-selectin and ICAM-1.

In order to evaluate the efficacy of NGF antisense ODN, acetic acid infusion was used to cause a rapid rise of NGF protein levels. A single dose instillation of OND complexed with liposomes protected against bladder overactivity (BO) induced by acetic acid. Together with data of bladder uptake studies using fluorescent ODN, it is demonstrated that OND is readily available to the bladder after intravesical instillation. It has been previously reported that, within 2 h of exposure to irritants such as turpentine and acetic acid, there occurs a rapid rise in bladder content of NGF [75]. Later studies found that bladder responds to insults with upregulation in the genes for NGF, sE-Selectin and receptor for monocyte chemoattractant protein-1 (MCP-1) within 30 min of exposure to lipopolysaccharide (Figure 5) [76]. The acetic acid induced overexpression of NGF was blunted by pretreatment with NGF antisense OND with phosphorothioate linkage prior to exposure of acetic acid. The downregulation of NGF mRNA expression was in agreement with reduced protein levels and suppressed NGF-like immunoreactivity in the urothelium.

### 9.3. Antisense and Downstream Signalling of NGF

Drug development of intravesical antisense for NGF was also able to unmask the downstream signaling [77] involving NGF following exposure to acetic acid. Experiments supported the role of NGF as a paracrine messenger [77], which is known to activate several downstream effectors to manifest physiological and pathological signaling changes linked to it [76, 78–80]. The bladder injury set off by acetic acid initiates the signalling cascades that upregulate the expression of NGF and other chemokines, MCP-1, CXCL-1, and CXCL-10, and prostaglandins [76, 78–80]. As reported elsewhere, localization of chemokines within synaptic vesicles in neurons [81] is consistent with their ability to act as excitatory neurotransmitters following AA exposure.

Chemokines are one of several downstream effectors activated by NGF [76, 78–80] and interestingly chemokine receptors are widely expressed in neural and nonneural elements of the nociceptive pathways that are responsible for visceral and somatic pain sensation [82]. MCP-1 [83] and CXCL-10 [84] are constitutively expressed in neurons, where they participate in excitability of primary afferent neurons through transactivation of transient receptor channels and nociceptor sensitization [84]. Overexpression of NGF is likely to drive the expression of VEGF from neurons and leptin [78] from adipocytes covering neurons [85].

Cooperative expression of NGF and MCP-1 is able to induce hyperexcitability in neurons by activating TRPV1 receptor. In addition, MCP-1, CXCL-1, and CXCL-10 cause chemoattraction of monocytes, neutrophils, and lymphocytes, respectively, to mediate the bladder injury set off by acetic acid. Extravasation is an essential prerequisite for infiltration of monocytes, neutrophils, and lymphocytes, which requires the expression of adhesion molecules like E-selectin and intracellular adhesion molecule ICAM-1 [76, 86]. E-selectin is a cytokine-inducible adhesion molecule that supports the rolling and stable arrest of leukocytes on activated vascular endothelium. Expression of E-selectin has been linked to adherence of neutrophils to bladder microvascular endothelial cells and to cyclophosphamide cystitis [87, 88]. E-selectin gene expression is activated by the NF-*κ*B and MAP kinase signal transduction pathways [76, 86].

Activation and recruitment of leukocytes to acetic acid induced bladder injury also require expression of intracellular adhesion molecule ICAM-1 [89]. ICAM-1 interacts specifically with its receptors of the integrin family to induce reversible cell-cell interactions involving adhesion. Expression of ICAM-1 was found to be increased in patients with IC/PBS and reduced in patients responding to instillation of hyaluronic acid [90]. Expression of ICAM-1 and VCAM-1 was also noted in a recent study on biopsy tissue of IC/PBS patients [91]. Increased expression of ICAM-1 subsequent to exposure with acetic acid in animal model is therefore clinically relevant.

Antisense experiments supported the earlier report that inhibition of NGF expression significantly downregulates the expression of ICAM-1 [80]. It is reported that binding of NGF to its high-affinity TrkA receptor controls the sICAM-1 expression on target cells [80]. Expression of ICAM-1 was negatively associated with NGF expression in antisense experiments. Ultimately, expression of ICAM-1 is regulated at the level of transcription by one of several signaling cascades NF-*κ*B, JAK/STAT, IFN-g (CXCL-10), AP, MAP kinase, and PKC [92].

Intravesical route can allow selective exposure of antisense ODN to the NGF producing cells in urothelium and avoid systemic side effects from genetic manipulation of NGF expression. Intravesical route can also prove to be cost effective given the cost of ODN or siRNA. Contrary to the goal of loss of function in bladder with antisense ODN, in recent studies liposomes have also been used to deliver ODN for gain of function in bladder [93].

## 10. Imaging of Intravesical Therapy

Drug development for DO and IC/PBS relies heavily on subjective outcomes for predicting efficacy and safety in early clinical development. Although there are many validated measurement tools used in DO and IC/PBS research, they are usually burdensome and do not capture all symptoms related to the lower urinary tract. Moreover, improvement in symptoms scores following an intervention does not always correlate with patient expectations, satisfaction, and goal achievement, which are critically important for successful management of DO and IC/PBS.

There is underdevelopment and underutilization of urine biomarkers and imaging methods for investigational drugs given by intravesical route. Overreliance on subjective impressions of patients for therapeutic response limits clinical relevance due to weak correlation with patient satisfaction and can also impede continued scientific progress in assessing study outcomes relative to symptom bother. Symptom scores may also be related to the cause of high heterogeneity in clinical response in DO and IC/PBS patients.

Imaging can offer the possibility to provide region-specific information in situations where serum and urine assays may not reflect the true state of bladder [94–96]. Urine chemokines have been reported to be associated with symptom severity of IC/PBS patients [97]. Application of fluorescent microscopy for imaging of pelvic floor has been limited due to the issues pertaining to light absorption by tissues, scattering, and autofluorescence [98].

Recent studies have shown that dyes fluorescing in the near-infrared (NIR) band can overcome this handicap in deep-tissue imaging of experimental animals [99]. Selected NIR fluorochromes emit light with tissue penetration approaching 10–15 cm [100]. The light emission from probes in this spectrum encounters low background autofluorescence and minimal attenuation of signal due to light absorption by tissue components [98]. Therefore, NIR imaging yields high signal-to-noise ratios and is well suited for studying the distribution of instilled treatments in bladder (Figure 2(b)).

Preliminary studies suggest that imaging of mouse pelvic floor in NIR spectrum can be a viable option [101]. NIR imaging easily allows visualization and quantification of bladder distribution by tracing the migration of fluorescence-labeled liposomes in anaesthetized mouse. Compared with other biological assays, imaging could provide objective endpoint for patients with ulcerative cystitis to indicate its high translation potential. Other benefits of optical imaging include its relatively low cost compared with more traditional imaging systems such as MRI and PET. The instrument consists of two near-infrared lasers emitting at 685 nm and 785 nm, camera, and novel optics.

Furthermore, preferential binding of liposomes and nanoparticles to bladder lesions of IC/PBS after instillation can be assessed by NIR imaging of bladder, where it can theoretically provide anatomically specific information about the real time status of ulcer/lesion in bladder surface or tissue abnormality [102]. Therefore, future studies can substitute a response variable that is continuous in nature instead of a subjective clinical outcome.

## 11. Conclusions

Adoption of forward-thinking approaches can achieve advancements in drug delivery systems targeted to improve pharmacotherapy of bladder diseases in the future. Latest developments in the field of nanotechnology can bring this mode of therapy from second line of treatment for refractory cases to the forefront of disease management. Liposomes are an attractive drug delivery platform by virtue of their biodegradability, biocompatibility, low toxicity, and simple and mild preparation methods.
